# Clinical Validation of Integrated Point-of-Care Devices for the Management of Non-Communicable Diseases

**DOI:** 10.3390/diagnostics10050320

**Published:** 2020-05-19

**Authors:** K. V. Giriraja, Suman Govindaraj, H. P. Chandrakumar, Basanth Ramesh, Licy Prasad, B. R. Priyanka, Samreen Shaikh, Varun Rana, Varun Trivedi, Sridhar Ramanathan, Ramanan Laxminarayan, Radha Rangarajan

**Affiliations:** 1 Rajalakshmi Hospital, 21/1, Lakshmipura Main Road, Vidyaranyapura Post, Bengaluru, Karnataka 560097, India; drgirirajkv@gmail.com (K.V.G.); drsumanraj6@gmail.com (S.G.); 2 HealthCubed India Private Limited, IKP-EDEN, Tavarekere Main Rd, Koramangala, Bengaluru, Karnataka 560029, India; chandrakumarhp@gmail.com (H.P.C.); basanth.ramesh@healthcubed.com (B.R.); licy.rp@healthcubed.com (L.P.); brpriyanka6@gmail.com (B.R.P.); samreen.shaikh@healthcubed.com (S.S.); varun.rana@healthcubed.com (V.R.); varun.trivedi@healthcubed.com (V.T.); sridhar.ramanathan@gmail.com (S.R.); rlaxmin@healthcubed.com (R.L.)

**Keywords:** point-of-care, integrated device, primary care, HealthCube-SE, HealthCube-XL, cardiovascular diseases

## Abstract

Non-communicable diseases are the leading cause of death and disability across India, including in the poorest states. Effective disease management, particularly for cardiovascular diseases, requires the tracking of several biochemical and physiological parameters over an extended period of time. Currently, patients must go to diagnostic laboratories and doctors’ clinics or invest in individual point-of-care devices for measuring the required parameters. The cost and inconvenience of current options lead to inconsistent monitoring, which contribute to suboptimal outcomes. Furthermore, managing multiple individual point-of-devices is challenging and helps track some parameters to the exclusion of others. To address these issues, HealthCubed, a primary care technology company, has designed integrated devices that measure blood glucose, hemoglobin, cholesterol, uric acid, blood pressure, capillary oxygen saturation and pulse rate. Here we report data from clinical studies undertaken in healthy subjects establishing the validity of an integrated device for monitoring multiple parameters.

## 1. Introduction

Non-communicable diseases (NCDs) are now the leading cause of disease burden in India, including in the poorest states. According to the World Health Organization, NCDs accounted for 63% of all deaths in India in 2016 with cardiovascular diseases (CVDs) contributing a major part of the burden (27%), followed by respiratory diseases (11%) [1]. CVDs include coronary heart disease, cerebrovascular disease, rheumatic heart disease and other conditions. Multiple studies show that CVDs present at earlier ages in India than in developed countries but often remain undiagnosed, leading to increased morbidity and mortality [2,3,4]. 

The management of NCDs, particularly CVDs, requires the monitoring of several parameters such as blood pressure, blood glucose and cholesterol. This necessitates multiple visits, to provide a blood sample, receive the report and consult with the physician. Given the long-term nature of the conditions, testing and monitoring has to be undertaken for years. Cost and inconvenience add up; moreover, the lack of high-quality diagnostic laboratories impedes the effective management of these diseases. To address these challenges, point-of-care (POC) tests that can be administered at the site of interest such as a clinic or a home, are rapid, easy to perform, and enable treatment decisions or referrals within a single encounter with the patient, have been introduced [5,6,7]. However, patients and caregivers must buy several devices, one for each parameter. For example, people who are diagnosed with diabetes must be monitored, not just for blood glucose levels, but also for cholesterol and blood pressure due to a higher risk of cardiovascular disease [8]. The inconvenience and cost to the patient and the clinician contribute to inconsistent or incomplete monitoring.

HealthCubed (HC) has designed a set of devices that measure blood glucose, hemoglobin, cholesterol, uric acid, blood pressure, capillary oxygen saturation and pulse rate, for the simultaneous monitoring of biochemical and physiological parameters relevant to CVDs. They are compact and lightweight (1.7 kgs) and are designed for convenience, ease of use, portability and accuracy. The devices are controlled by an android software application via Bluetooth and offer data storage on the cloud. These features make the devices valuable screening tools in the hands of a healthcare provider such as a general practitioner, particularly in semi-urban and rural areas. 

While other devices with multiple test offerings exist (Table 1; Comparison of integrated point-of-care devices), none offer as comprehensive a set as the HC devices. In particular, having biochemical and physiological tests on the same platform is unique. Further, cloud-based storage of data through the HC devices enables long-term monitoring of patients.

The HC devices are an aggregate of POC tests/devices, each of which has been individually validated by its manufacturer and approved for use in India. To confirm the performance characteristics of HealthCube-SE (HC-SE) and HealthCube-XL (HC-XL) in comparison with individual, comparable POC tests/devices, we undertook a clinical study in healthy subjects. Here we report results demonstrating the validity of these integrated devices.

## 2. Materials and Methods

### 2.1. Study Design

We evaluated the performance characteristics of point-of-care devices, HealthCube SE (HC-SE) and HealthCube XL (HC-XL) compared to equivalent marketed devices. HC-SE was evaluated for blood glucose, hemoglobin, cholesterol, uric acid and HC-XL was evaluated for systolic and diastolic blood pressure, peripheral capillary oxygen saturation and pulse rate. HC-SE has the same functionalities as HC-XL, with the exception of the electrocardiography module.

The study was conducted by the Rajalakshmi Hospital, Bengaluru, India, a tertiary care hospital approved by the Central Drug Standards Control Organization (CDSCO) for clinical validations. The study was open label, outcome blinded and conducted in accordance with the Declaration of Helsinki. It was approved by the institution’s Ethics Committee prior to commencement under approval number RH/IEC/AP-024/2018 (approved on 17 September 2018) for HC-SE and RH/IEC/AP-023/2018 (approved on 17 September 2018) for HC-XL. The study was registered under Clinical Trials Registry–India (CTRI) with the number CTRI/2019/01/016905 (approved on 4 January 2019). It was performed in 2 phases; in the first phase, tests for blood glucose (BG), hemoglobin, cholesterol and uric acid were conducted using HC-SE (Study 1). In the second phase blood pressure and pulse oximetry were evaluated using HC-XL (Study 2).

### 2.2. Participants

Eligible participants were healthy volunteers who were recruited to the study between 11 October 2018 to 15 April 2019. A cohort of 150 subjects participated in Study 1 and subsequently in Study 2. The inclusion criteria were adults, 18 years and above who were able to give consent. The exclusion criteria were known diagnosis of acute or critical illness, participation in any other clinical study and history of blood disorders such as lymphoma, leukaemia, myeloma, polycythaemia, Von Willebrand disease, haemophilia and thrombocythemia. The participants were 70.7% male and 29.3% female. All participants gave informed consent.

### 2.3. Study Procedures

#### 2.3.1. Study 1

Each participant was subjected to a fingerstick to obtain capillary blood and tested alternately on the reference device and the HC-SE device. The reference devices were CE certified, BeneCheck Multimonitoring System (BK6-12M) for glucose, cholesterol and uric acid and CE certified Premium Hb monitoring System (BKA-30S) for hemoglobin. The data were recorded for the reference devices manually. The HC-SE device provided a readout on a Bluetooth-enabled tablet device through an android application called EzDx for Easy Diagnostics (version 2.6 demo).

#### 2.3.2. Study 2

(a) Blood Pressure: Each participant was tested while seated and resting with the FDA approved SunTech 247 NIBP Model 551503 (reference device) and HC XL. Three measurements were taken on each device by the same operator, alternating between the devices with a 1-minute gap between readings. The measurements were manually noted and averaged.
(b) Pulse oximetry: Peripheral capillary oxygen saturation and pulse rate were measured using FDA approved Choicemmed MD300C2 Pulse Oximeter (reference device) and HC XL using a probe connected to the subject’s index finger. Measurements were taken in 3 replicates, alternating between the devices. Averages were calculated and compared.

### 2.4. Data Analysis

#### 2.4.1. Study 1

The results obtained for blood glucose, total cholesterol, uric acid and hemoglobin using the HC-SE device were compared with the respective reference device readings. Standard statistical techniques, such as proportions that fit within specific limits (acceptable tolerance limits), were applied (Table 2). These limits were based on the disclosures of the manufacturers of the individual POC devices that are integrated within HC-SE [9,10,11] and point-of-care industry standards [12].

Scatter plots were drawn and the Pearson correlation coefficient was derived to describe the relationship between data obtained from the HC-SE device and the reference device.

#### 2.4.2. Study 2

Systolic and diastolic blood pressure values, oxygen saturation and pulse rate were compared between the HC-XL device and the reference device. Proportions that fit within specific limits were determined for each parameter (Table 3). Like in study 1, these limits were based on manufacturers’ disclosures and industry standards [13]. Scatter plots were generated and Pearson correlation coefficient values were calculated as for Study 1.

## 3. Results

### 3.1. Study 1

**Figure 1:**
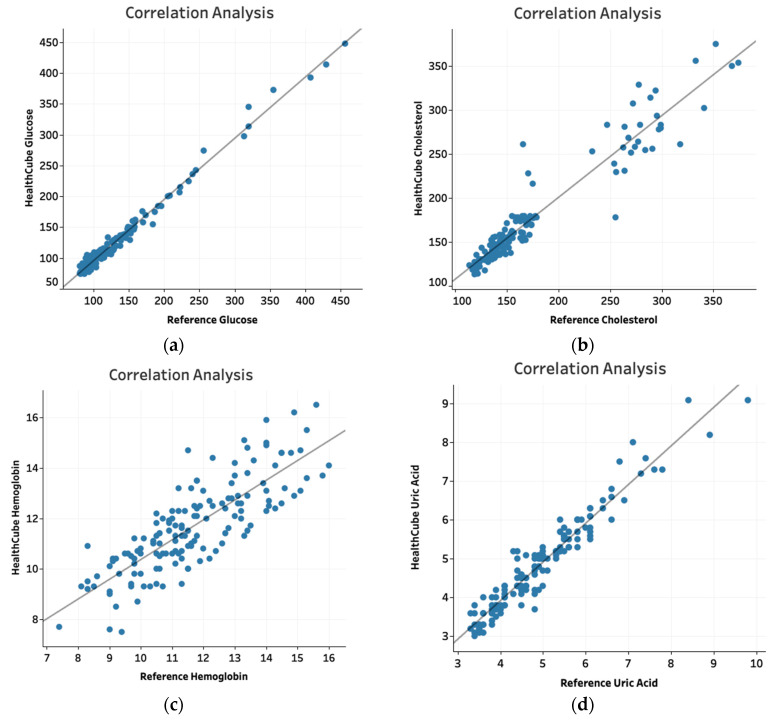
Relationship between HC-SE values and reference device values depicted in scatter plots for glucose (**a**), cholesterol (**b**), hemoglobin (**c**) and uric acid (**d**).

In Study 1, biochemical parameters were compared between the HC-SE and reference devices. Of the 150 subjects recruited for the study, 150, 148, 146 and 143 subjects were evaluated for blood glucose, total cholesterol, hemoglobin and uric acid, respectively (Table 4). Patients from whom inadequate blood was obtained were excluded. For blood glucose, data for 41/43 (95.3%) subjects were within ±15 mg/dL for values < 100 mg/dL and 103/107 (96.3%) were within ±15% for levels ≥ 100 mg/dL compared to data from the reference device. For total cholesterol, data for 73/73 (100%) subjects were within ±20 mg/dL for values < 150 mg/dL and data for 71/75 (94.7%) subjects were within ±20% for cholesterol levels ≥ 150 mg/dL compared to reference device values. For hemoglobin, data for 137/146 (93.8%) subjects were within ±15% compared to reference device values. For uric acid, data points for 82/82 (100%) subjects were within ±1.5 mg/dL for values < 5 mg/dL and data points for 61/61 (100%) subjects were within ±20% of reference device values for levels ≥ 5 mg/dL. Scatter plots generated for each parameter demonstrated Pearson correlation coefficient values of 0.99 for Glucose, 0.96 for Cholesterol, 0.88 for hemoglobin and 0.96 for uric acid (Figure 1a–d).

### 3.2. Study 2

**Figure 2:**
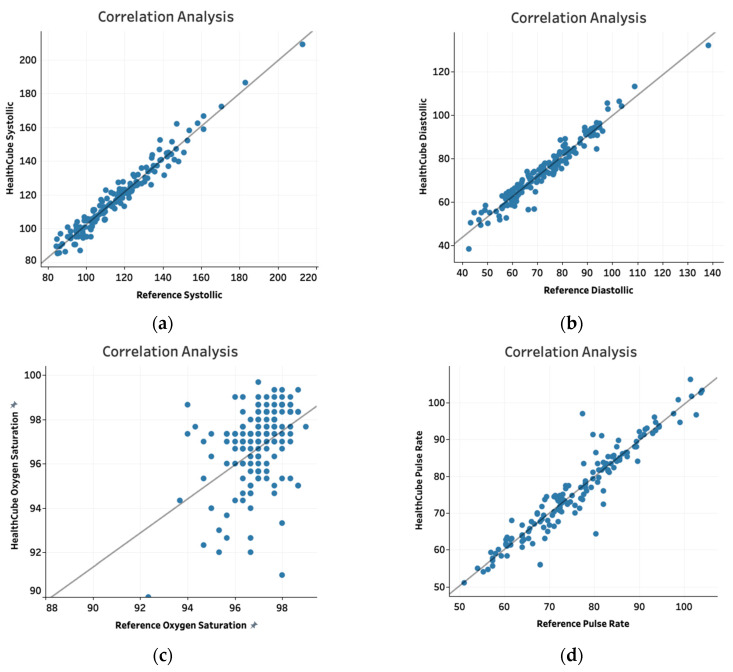
Correlation between HC-XL and reference device values depicted in scatter plots for systolic pressure (**a**), diastolic pressure (**b**), oxygen saturation (**c**) and pulse rate (**d**)

Blood pressure, oxygen saturation and pulse rate measured with the HC-XL device were compared with measurements from the appropriate reference devices (Table 5). 143/150 (95%) data points were within ±10 mm Hg for systolic blood pressure and 148/150 (98.7%) data points were within ±10 mm Hg for diastolic blood pressure compared to data from the reference device For the pulse oximetry module, data for 144/150 subjects (96%) were within ±4% of reference device values for oxygen saturation and 144/150 (96%) were within ±6 beats per minute (bpm) for pulse rate. Scatter plots for blood pressure and pulse oximetry values showed correlation coefficients of 0.98 and 0.97 for systolic and diastolic blood pressure, respectively, and 0.42 and 0.95 for oxygen saturation and pulse rate, respectively (Figure 2a–d).

## 4. Discussion

We evaluated the performance characteristics of the HC-SE and HC-XL devices compared to reference devices for glucose, cholesterol, hemoglobin, uric acid, systolic and diastolic pressure, pulse rate and oxygen saturation. The tests were evaluated in two ways. One, the values obtained with the HC devices were compared to reference device values and binned into ranges based on specific tolerance limits. Two, scatter plots were drawn and the correlation between HC and reference device values was analyzed.

For all parameters except cholesterol and hemoglobin, more than 95% of the data points fit within acceptable tolerance limits. Of the data points for cholesterol and hemoglobin, 94.6% and 93.8%, respectively, were within acceptable tolerance limits. Furthermore, the Pearson correlation coefficient values were 0.99 for glucose, 0.96 for cholesterol, 0.88 for hemoglobin and 0.96 for uric acid. For blood pressure, pulse rate and oxygen saturation, the Pearson coefficient values were 0.98 for systolic blood pressure, 0.97 for diastolic blood pressure, 0.42 for oxygen saturation and 0.95 for pulse rate, respectively. The Pearson coefficient values for all parameters except hemoglobin and oxygen saturation, were >0.9, suggesting an excellent correlation between the HC and reference devices.

The HC-SE and HC-XL devices are intended for screening purposes. Their accuracy needs to be similar to other point-of-care or home-based use devices. Moreover, since the HC devices are aggregates of individual point-of-care tests, the performance characteristics of each test must be in line with disclosures made by the manufacturers for those tests. These expectations were largely met, with inconsistencies noted for cholesterol, hemoglobin and oxygen saturation. The cholesterol test data, although borderline in the range analysis, had an excellent correlation to the reference device. Hence, this test meets one of the key validation criteria. The hemoglobin test appeared to be less robust, both in the range and correlation analysis. However, this test may offer a reasonable estimation in clinical settings where diagnostic laboratories are hard to come by. For oxygen saturation, we note that 96% of the values fell within the allowed range of variability, even though a poor correlation between device values was observed. Since the comparison was between two point-of-care devices, factors such as the calibration of the reference device cannot be ruled out as the cause of the poor correlation. Additional studies comparing HC device values with invasive venous blood oxygen saturation measurements may be needed to confirm the accuracy of this test.

One limitation of this study was that it was conducted in healthy subjects. Thus, the spread of values was limited, with the exception of hemoglobin. Given the high incidence of anaemia in India, this is not a surprise [14]. To ensure a more comprehensive validation, future studies should aim to cover a more complete range for each parameter.

The HC-SE and HC-XL devices allow users to obtain data, for several key parameters using a single, integrated device. For clinicians and caregivers in diverse settings, this eliminates the need to invest in multiple devices and train individuals to operate them. It provides convenience and importantly, allows clinicians to make informed decisions for their patients. These are significant benefits, particularly in underserved communities that have poor access to diagnostic laboratories. We believe that the HC devices push the frontiers of disease screening and diagnosis, addressing directly the need for managing chronic conditions in India and other parts of the world equitably.

## Figures and Tables

**Table 1:** Comparison of integrated point-of-care devices.

Name of the Device	Features	Disadvantages
Checkme™ Lite Vital Signs Monitor	Measurement of ECG, Spo2 and Systolic blood pressure	Does not measure biochemical parameters
HumaSensplus	Measures Glucose, Uric Acid, Total Cholesterol	Does not measure vitals
CardioChek Home Basic Analyzer	Measures total cholesterol, HDL triglycerides, glucose	Does not measure vitals

**Table 2:** Tolerance limits for blood glucose, cholesterol, hemoglobin and uric acid.

Test	Cutoff Value	Acceptable Tolerance Limits
Blood Glucose	<100 mg/dL	±15 mg/dL
	≥100 mg/dL	±15%
Cholesterol	<150 mg/dL	±20 mg/dL
	≥150 mg/dL	±20%
Hemoglobin	7 to 26 g/dL	±15%
Uric Acid	<5 mg/dL	±1.5 mg/dL
	≥5 mg/dL	±20%

**Table 3:** Tolerance limits for blood pressure and pulse oximetry.

Test	Parameter	Clinically Observed Range	Acceptable Tolerance Limits
Blood Pressure	Systolic	40–250 mmHg	±10 mm/Hg
	Diastolic	30–190 mmHg	±10 mm/Hg
Pulse Oximetry	Oxygen Saturation	35–100%	±4%
	Pulse Rate	25–250 bpm	±6 bpm

**Table 4:** Fit of data within specific ranges for blood glucose, cholesterol, hemoglobin and uric acid.

Test	Cutoff	Tolerance Limits	Total Subjects	Number within Limits	Percentage within Limits
Blood Glucose	<100 mg/dL	±15 mg/dL	43	41	95.3
	≥100 mg/dL	±15%	107	103	96.3
Cholesterol	<150 mg/dL	±20 mg/dL	73	73	100
	≥150 mg/dL	±20%	75	71	94.7
Hemoglobin	7 to 26 g/dL	±15%	146	137	93.8
Uric Acid	<5 mg/dL	±1.5 mg/dL	82	82	100
	≥5 mg/dL	±20%	61	61	100

**Table 5:** Fit of data within specific ranges for systolic and diastolic pressure, oxygen saturation and pulse rate.

Test	Parameter	Clinically Observed Range	Tolerance Limits	Total Subjects	Number within Limits	Percentage within Limits
Blood Pressure	Systolic	40–250 mmHg	±10 mm/Hg	150	143	95.3
	Diastolic	30–190 mmHg	±10 mm/Hg	150	148	98.7
Pulse Oximetry	Oxygen Saturation	35–100%	±4%	150	144	96
	Pulse Rate	25–250 bpm	±6 bpm	150	144	96
